# A statistical analysis of causal factors influencing college student’s willingness to consume digital music

**DOI:** 10.1371/journal.pone.0324168

**Published:** 2025-06-02

**Authors:** Yu Shan, Fengdie Hu, Yabo Xie, Lixin Bai, Eksiri Niyomsilp

**Affiliations:** 1 School of Music and Dance, Xihua University, Chengdu, China; 2 School of Management, Shinawatra University, Pathum Thani, Thailand; 3 School of Management, Southeast Asia University, Bangkok, Thailand; 4 College of Water Resource and Hydropower, Sichuan University, Chengdu, China; Rikkyo University: Rikkyo Daigaku, JAPAN

## Abstract

Determining the causal factors influencing college students’ willingness to consume digital music in China is crucial given the rapid integration of digital technology with the traditional music industry. Chengdu, as a rapidly developing city in China with a thriving youth culture and a significant presence of higher education institutions, provides an ideal setting to explore the factors influencing college students’ willingness to consume digital music. Using a mixed-method approach and a sample of 431 college students from various universities in Chengdu, this research examines the impact of perceived value, behavioral attitude, subjective norms, user participation, user stickiness, and psychological needs on digital music consumption intention. The empirical results show that these factors jointly affect consumption intention, with user stickiness and psychological needs serving as mediators. Specifically, perceived value, behavioral attitude, subjective norms, and user participation have a significant positive impact on consumption intention. These findings provide valuable insights for digital music platforms and the music industry to develop targeted marketing strategies and services tailored to the demands and behaviors of college students in Chengdu.

## 1. Introduction

Digital music refers to the type of music that is saved and transmitted using advanced digital technology. It can achieve efficient transmission and sharing of music through the Internet and wireless networks. Compared to traditional music, digital music has the characteristics of being easier to download, copy, and play, and while retaining high-quality sound quality, it can also provide a more flexible and autonomous digital experience for music fans [1]. This has enabled digital music to achieve excellent performance in terms of speed, convenience, fashion, and has become one of the popular ways of music dissemination in today’s society. China’s digital music industry enjoys strong development momentum. The 2023 Global Music Report by the International Phonographic Industry reveals that China has ascended to the position of the fifth largest music market in the world. Overall, streaming remains the core distribution method for video and music, driven by the growth of streaming. The streaming market is still leading the rapid development of the entire digital music industry [2]. China, the second largest market in Asia, saw a growth rate of 28.9 percent. It can be seen that China’s digital music market is sizeable and has broad space for development. In the era of mobile Internet, the payment consciousness of Internet digital music streaming media users has increased significantly. The post-90s and post-00s generation has become the dominant group of digital music consumption, and their willingness to pay for quality content services is becoming increasingly important.

Against this backdrop, the following questions naturally arise: What factors influence the willingness of young people, particularly college students, to consume digital music? Do these factors interact with one another? And how can targeted marketing strategies be formulated based on the findings? Unraveling the influencing factors and their interactions regarding the willingness of young people, especially college students, to consume digital music holds not only academic significance in gaining a deeper understanding of this demographic’s consumption behavior but also provides practical guidance for music platforms to devise targeted marketing strategies, enhance user experiences, and foster the healthy development of the music market.

In 2023, the scale of China’s digital music industry will reach 89.345 billion yuan, a year-on-year increase of 5%, with a slight slowdown in growth rate. The number of online music users has reached 726 million, accounting for 67.6% of the total number of netizens [2]. With the gradual improvement of the copyright protection system and the continuous growth of users’ demand for high-qualimusic content, the number and payment ratio of paying users on major digital music platforms have both experienced double-digit growths.

The rise of streaming platforms is reshaping the global entertainment industry at an astonishing speed. Over the past decade, the streaming industry has witnessed explosive growth, giving birth to many leading companies. By mid-2023, China’s online video users had surpassed 1 billion, becoming a significant force in the global video streaming market. Among them, iQiyi, Youku, and Tencent Video have stood out as major players. Meanwhile, Southeast Asia has become the fastest-growing Internet economy. The COVID-19 pandemic restricted people’s movements but also accelerated the development of streaming services, with mobile streaming users in Southeast Asia increasing by 60%. An increasing number of streaming services and content providers have begun to enter the market, such as Netflix, Disney + , and TikTok. The development of digital music and streaming media platforms is closely linked. The rise of digital music provides a rich content foundation for streaming media platforms, while streaming media platforms promote the popularity of digital music through convenient online playback and personalized recommendations. Both have jointly changed users’ music consumption habits, shifting from physical purchases to online listening and subscription services. In addition, streaming media platforms have further promoted the digital transformation and commercial development of the music industry through data analysis and copyright management. The rise of digital music streaming, widespread social media usage, and the increasing prominence of big data and algorithms in music recommendation systems have fundamentally transformed music consumption patterns and motivations [3]. These changes not only impact consumers’ choices and experiences but also reshape the ecosystem for artists, producers, and the entire music industry. In a complex market environment, online videos exert a multifaceted influence on recorded music sales, encouraging users to actively explore music on social platforms [4]. This tendency towards seeking inspiration and social connections has turned music consumption into a social activity, especially with the emergence of short video platforms like TikTok, where users broaden their musical horizons through creation and sharing [5].

Within this context, the digital environment has fueled consumers’ desire for diversity and personalization, leading to a reevaluation of traditional music consumption models and necessitating novel understandings [6]. The rapid evolution of digital technology has garnered widespread attention regarding shifts in music consumption motivations and behaviors. Existing research highlights significant changes in how consumers acquire and consume music, profoundly affecting their motivations and behavior patterns.

In the German market, online videos’ impact on recorded music sales is intricate, influenced not only by platform availability but also by social interaction, individual preferences, and cultural backgrounds [4]. Further exploration of user motivations on digital music platforms reveals that users seek not just personal entertainment but also inspiration and social connections (Nunes and Birdsall, 2022). Consequently, the surge in digital music consumption has fostered an openness to new music among consumers and prompted reflections on traditional consumption patterns, indicating a growing public emphasis on music diversity and innovation in the digital age [6]. An essential topic for the further development of digital music is understanding the factors that influence consumers’ choices in digital music consumption. Notably, personalized needs and convenient experiences significantly impact consumer decisions [7]. Meanwhile, the socio-cultural background plays a crucial role in shaping consumers’ digital music consumption motivations, assigning music diverse meanings across different cultural or social groups [8]. Consumers’ music choices mirror not only their preferences for notes and melodies but also their identity [9]. Therefore, the evolution of music consumption motivations is not solely a direct consequence of digital technology development but also reflects the intricate interplay of social interaction, cultural identity, and personal experience. Among teenagers, using music sharing to construct personal images and seek recognition and support from peers has become a prevalent phenomenon [10].

From the perspective of user structure, college students are the leading group of music consumption although they have less income. China Mobile’s digital music paying users are mainly low-income students and high-income professional elites. College students, representing the younger demographic, have matured alongside the evolution of the Internet and are more likely to have a typical relationship with music. In particular, the post-90s and post-00s are the major users and fans of digital music platforms, and their paying power cannot be underestimated. Moreover, college students represent a critical demographic for digital music platforms due to their unique behavioral and socio-cultural characteristics. First, as “digital natives” who have grown up in the internet era, they exhibit higher adaptability to technological innovations and digital consumption patterns compared to older generations [8]. This makes them early adopters of digital services, providing insights into future market trends. Second, despite their limited disposable income, their consumption behavior is heavily influenced by social dynamics, which aligns with the study’s focus on subjective norms and user participation [9]. Additionally, college students’ music consumption habits are often intertwined with identity construction and cultural expression, making them a strategic group for understanding the interplay between psychological needs and consumption intentions [10]. Finally, their concentrated presence in urban educational hubs like Chengdu offers logistical advantages for targeted data collection while reflecting regional market dynamics[11]. By focusing on this cohort, the study captures both the immediacy of youth-driven digital consumption and its implications for broader industry strategies.

As an important supporting part of the booming development of China’s digital music industry, the construction of regional music industry bases not only provides strong support and guarantee in many key aspects such as hardware facilities, resource integration and sharing, talent cultivation and education, and market expansion, but also firmly builds the development cornerstone of the core links of the digital music industry chain [11]. As mentioned earlier, although previous studies have investigated people’s willingness to consume digital music from various perspectives, these studies lack systematicity and the research subjects are not focused enough. Therefore, based on previous research, this article systematically studies the influencing factors of digital music consumption willingness among college students in Chengdu, China. The theoretical framework for consumer behavior analyzed statistically in this paper encompasses four dimensions: perceived value, behavioral attitude, subjective norm, and user involvement.

1. When making consumption decisions, consumers comprehensively consider factors such as product value, price, and quality. Products with high perceived value are more likely to gain favor among consumers. Existing research has shown that perceived value is a significant factor influencing consumers’ purchase intention (Consumer Perceived Value, Involvement, Trust, Susceptibility to Interpersonal Influence, and Intention to Participate in Online Group Buying).
2. Consumers’ attitudes toward a product influence their purchase intention. Positive behavioral attitudes promote purchase behavior, while negative attitudes inhibit it. Studies have indicated that behavioral attitude is a crucial factor affecting consumers’ purchase intention (A New Model to Predict Consumers’ Willingness to Buy Fair-Trade Products; Consumer Antecedents Towards Green Product Purchase Intentions).
3. Consumers’ purchase behavior is influenced by those around them. When a product is widely recognized by others, consumers are more likely to develop purchase intention. Research has demonstrated that subjective norm is a significant factor influencing consumers’ purchase intention (The Roles of Values and Social Norm on Personal Norms and Pro-Environmentally Friendly Apparel Product Purchasing Behavior: The Mediating Role of Personal Norms).
4. User involvement refers to the effort and engagement invested by users during product usage. Users with high involvement are more likely to develop a sense of identification and loyalty to the product, leading to purchase intention. Studies have shown that user involvement is an important factor affecting consumers’ purchase intention (Consumer Engagement in Social Media Brand Communities: A Literature Review). Perceived value is a well-established construct in consumer behavior research, particularly in the context of digital goods and services. In the context of digital music, perceived value is crucial because college students, who often have limited disposable income, are likely to weigh the benefits of paid music services against their costs. We chose this variable because it directly addresses the cost-benefit analysis that college students are likely to perform when deciding whether to pay for digital music. In the context of digital music consumption, behavioral attitude captures students’ perceptions of the ethical and practical aspects of paying for music. Given the historical prevalence of free music consumption on the internet, understanding how attitudes toward paying for music influence consumption decisions is critical. We included this variable to explore how students’ attitudes toward paid music services impact their willingness to consume. In the context of college students, peer influence and social norms play a significant role in shaping consumption behaviors. We chose this variable because it captures the social dynamics that are likely to influence college students’ decisions to pay for digital music, especially in a collectivist culture like China. In the context of digital music, user participation can include activities such as creating playlists, sharing music, and interacting with other users on the platform. We included this variable because it reflects the active role that users play in shaping their own consumption experiences, which is particularly relevant for college students who are highly engaged with digital platforms. While other variables such as psychological needs and user stickiness are also relevant, they were included as mediating variables in our model rather than independent variables. This decision was based on the theoretical framework of the Technology Acceptance Model (TAM) and the Theory of Planned Behavior (TPB), which emphasize the importance of perceived value, attitudes, and social norms as primary drivers of behavioral intention. Based on the aforementioned theoretical foundations and research findings, this study proposes the following hypotheses:

H1: Perceived value has a significant positive impact on college students’ willingness to consume digital music.

H2: Behavioral attitude has a significant positive impact on college students’ willingness to consume digital music.

H3: Subjective norm has a significant positive impact on college students’ willingness to consume digital music.

H4: User involvement has a significant positive impact on college students’ willingness to consume digital music.

In 2016, the Opinions of the Chengdu Municipal People’s Government on Supporting the Development of the Music Industry “ guideline has been issued by the Chengdu municipal government, which clearly proposes the grand vision of building Chengdu into the music capital of China and even a globally renowned music city. Based on this, a series of targeted and effective related work have gradually been carried out. As of the end of 2021, the number of college students in Chengdu has reached 1.102 million, including 981000 undergraduate students and 120000 graduate students (including 12000 doctoral students). This has made Chengdu the city with the highest number of college students in southwestern China. The study on the influencing factors of digital music consumption willingness among college students in Chengdu will provide new ideas and research basis for theoretical construction in related fields, expand the framework of influencing factors of digital music consumption willingness, and provide empirical research results. At the same time, it will also provide reference for the study of regional differences.

On the basis of in-depth analysis and criticism of existing literature on the motivation and behavioral changes of digital music consumption, as well as the interaction between digital and traditional channels, this article conducts a unique study on the impact of college students’ willingness to pay for digital music consumption. Firstly, in line with the profound impact of digital technology on music consumption behavior emphasized in existing literature [4,5] used descriptive statistical methods to describe in detail the basic characteristics of college students in Chengdu, especially their preferences and habits in digital music consumption, providing an empirical basis for understanding music consumption behavior in specific regional cultural backgrounds. This approach not only enriches the geographical diversity of existing literature, but also enhances the pertinence and practicality of research.

Secondly, based on the summary of relevant research content, this article not only identifies the main factors affecting online digital music payment, but also constructs an innovative research model through questionnaire design, questionnaire survey, and data retrieval. This model not only incorporates core variables such as personalized needs and convenient experiences in digital music consumption [12], but also considers complex factors such as social interaction and cultural background. These factors have been widely discussed in existing literature, but research on their impact on specific payment intentions is still insufficient. Thirdly, the empirical analysis in this article draws unique conclusions based on critical reference to existing literature. In line with the existing literature [6] emphasizing that the increase in digital music consumption promotes consumers’ open attitudes towards new music, this article finds that college students exhibit a high pursuit of diversity and innovation in digital music consumption. However, unlike existing literature that mostly focuses on macro level trend analysis, this article reveals the specific manifestations of this pursuit among college students and the complex motivations behind it through detailed empirical analysis. In addition, this article also explores the potential impact of digital and traditional channel interaction on the willingness of college students to pay for music consumption [13], but focuses more on this specific consumer group, providing new evidence for understanding the role of multi-channel interaction in segmented markets.

In summary, this article conducts an in-depth analysis of the impact of college students’ willingness to pay for digital music consumption through empirical research and innovative research models. This study not only enriches the theoretical framework of digital music consumption behavior, but also provides targeted market strategy recommendations for music industry practitioners, especially in how to attract and cultivate the important consumer group of college students. The unique contribution of this article lies in its consideration of regional cultural backgrounds, analysis of the comprehensive effects of multiple factors, and exploration of the interaction between digital and traditional channels, providing a new perspective for understanding the complexity and diversity of music consumption in the digital age.

## 2. Methods

### 2.1. Sample and study design

We conducted a questionnaire survey of a sample of 431 college students in the China. Participants were sampled from Chengdu. As of May 2024, the total number of full-time students at Chengdu is over 41000. The survey questionnaire was approved on March 20, 2024, and the ethics review work was completed by the Ethics Committee of Xihua University (code XHA22403200055). The survey was distributed through online platforms such as ‘WeChat’ and ‘Wenjuanxing’. These platforms are widely used among Chinese college students. Participants aged between 16 and 24 voluntarily completed the survey after informed consent. Participants voluntarily click on the link to fill out the questionnaire. Before filling out the questionnaire, they were informed of the purpose of the questionnaire and informed that “submitting the questionnaire” is considered informed consent. Participants can withdraw at any time during the questionnaire filling process. For this survey, we sampled undergraduate students (16–24 years old) from all majors at Chengdu, including Business Administration, Software Engineering, Mechatronic Engineering, Materials Science and Engineering, Traffic Engineering, Energy and Power Engineering, Bioengineering, Pharmaceutical Engineering, Civil Engineering, Financial Management, Business Management, Accounting, Food Science and Engineering, Electrical Engineering and Automation, Information Engineering, Computer Science and Technology, Animation, Dance Performance, Fine Arts, Economics, Financial Technology, Physical Education, Law, Preschool Education, Chinese Language and Literature, Chemistry, Mathematics and Applied Mathematics, Psychology, Network and New Media, and so on.

The study selects university students in Chengdu as the research subjects primarily for the following reasons: Firstly, Chengdu, as a rapidly developing city in the southwestern region, boasts a vibrant youth culture and numerous higher education institutions, making the university student population representative. Secondly, the digital music industry in Chengdu is experiencing swift growth, and the consumption behavior of university students towards digital music exhibits certain typicality. Thirdly, this study aims to explore the factors influencing university students’ willingness to consume digital music. By selecting university students in Chengdu as the research subjects, it can better reflect the consumption characteristics of this demographic.

It must be acknowledged that, due to the sample being solely drawn from Chengdu, the generalizability of the research conclusions requires further validation. Future studies can broaden the sample scope to investigate the digital music consumption behavior of university students in other regions or countries, in order to test the generalizability of the research conclusions.

Additionally, we acknowledge that the existence of data bias is difficult to avoid. This study collects data through a questionnaire survey method, which may be subject to self-report bias, social desirability bias, and memory bias. In response to this, the following measures will be adopted to control for data bias: Firstly, anonymous questionnaire surveys will be conducted to protect the privacy of respondents and reduce social desirability bias. Secondly, a well-established scale will be utilized, with reliability and validity tests conducted to ensure data reliability and validity. Thirdly, questionnaire screening will be performed to eliminate invalid questionnaires and improve sample quality.

### 2.2. Survey instrument

**Fig 1:**
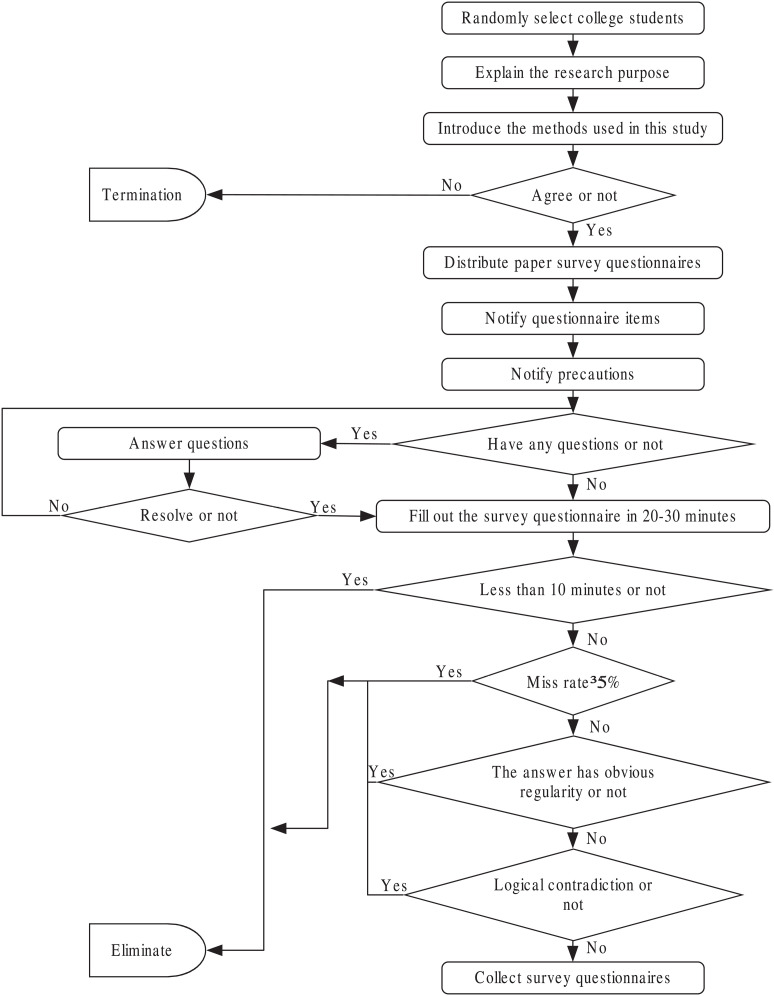
Data collection procedures.

The survey asked students about their preferences for consuming digital music, and other information like their economic situation as well as demographic information. The full survey information is provided in S1 and S2 Appendix. The author (Yu Shan) is a music teacher at Xihua University, with part-time teaching positions at Sichuan University of Culture and Arts and Geely University, where she delivers the course ‘Music Appreciation’. Teaching four classes of 150 students each semester provides convenient access to collect music-related questionnaires. The survey for this study was conducted in these Music Appreciation classes. The questionnaire is distributed randomly to college students. The questionnaire was distributed in electronic form for a limited time to ensure the quality of the questionnaire filling. The purpose of the study and the method used were explained in detail to the surveyed college students, and after getting the consent, the questionnaires were sent to them, and the questionnaire items and notes for filling were informed with unified guidelines. If there were any questions, the researchers would answer them in time, and the time for completing the questionnaire was controlled within 20–30 minutes. After the questionnaire is completed, the researchers will check the filling, remove unqualified questionnaires in time, and recover them uniformly. The exclusion criteria were: the questionnaire filling time was less than 10 minutes; Missing rate ≥5%; The answers to the questionnaire showed regularity; Inconsistent logic, or there are obvious errors in the questionnaire. If a questionnaire meets any of these criteria, it will be considered as an unqualified questionnaire as shown in Fig 1.

According to the research needs, 7 factors, such as perceived value, behavioral attitude, subjective norms, user participation, user stickiness, psychological demand and consumption intention, were selected as the variables of this study, and descriptive statistics were carried out on the 7 variables with relevant scales. Likert-type 5-point scoring method was used for the above scales, ranging from 1 “strongly disagree” to 5 “strongly agree”. Based on the basic principle of demographic profile and the survey characteristics, this study formulated 7 items as shown in Table 1, including gender, grade, average monthly consumption during school, source of living expenses, monthly expenses for digital music, living expenses, and spending methods.

**Table 1:** Measurement items for demographics.

Name	Options
Gender	male
	female
Grade	Freshman Year
	Sophomore year
	Junior
	senior
Average monthly spending during the school year	600-1000.
	1001-1500.
	1501-2000.
	Over 2000
Source of living expenses	All from family
	Partly from family, partly earned
	Earn it all by yourself
Monthly expenses for digital music	There is no
	1-200.
	201-500.
	500 +
You feel your living expenses	To have enough
	Just enough
	Not enough
The way of spending is	Plan it all before you spend it
	Save if you can
	Spend as much as you want
	Plan as you spend
	Other

### 2.3. Data analysis

The data analysis had two main components. First, we designed operational question items for all variables and formed a questionnaire with 42 question items. Second, based on this, the validity analysis (content validity - IOC test and structure validity - EFA) and reliability analysis were carried out for 36 variable data. Additional details are provided below. According to the previous assumptions, the study selected perceived value, behavioral attitude, subjective norms and user participation as independent variables of this study, and measured them with relevant scales. These four items were given in the form of multiple-choice questions. The settings and options of each item are shown in Table 2.

**Table 2:** Measuremens of independent variables.

Measurement Items	Topics
**Perceived Value**	I believe that the paid music business is worth buying and good value for money
	I think the paid music business makes it easier to listen to and download music
	Paid music allows me to enjoy more music resources
	Paid music allows me to enjoy a better audio-visual experience
	Paid music allows me to get more membership benefits
	Instead of switching between apps, buying paid music directly is more convenient, quality guaranteed and more cost-effective
**Behavioral attitude**	I think digital music charges are a result of compliance with copyright protection laws
	I think it is ethical to charge for digital music as a commodity
	Enjoying free music is a user habit cultivated by the Internet, which is difficult to change
	It is difficult to strengthen users’ awareness of music copyright and cultivate the habit of paying for music consumption
	I think charging for digital music will encourage musicians to create better music
**Subjective Norm**	More people around me understand my move to buy digital music
	More people around me supported the move to charge for digital music
	More people around have purchased paid music services (single album membership online concert)
	If my family or friends recommend me to buy paid music, I will try to buy it
**User participation**	I create different playlists for different types of music
	I follow and bookmark certain artists or music
	I like certain music when I listen to digital music
	I will participate in comments while listening to music on digital music platforms
	I interact and communicate with other users in the digital music community
	I share and forward some digital music to others
	I will post and repost information about digital music or artists on social networks or other platforms to communicate with others.

Consumption intention is selected as the dependent variable of this study to test the consumption intention of college students, and it is given in the form of multiple-choice questions. The items are set in Table 3.

**Table 3:** Measurements of dependent variables.

Measurement Items	Topics
**Consumption will**	If I come across paid music that I like, I will try to buy it
	I can see myself buying paid music in the future
	I would love to recommend good paid music to friends and family

User stickiness and psychological needs are selected as the mediating variables in this study, which are given in the form of multiple-choice questions. The item Settings are shown in Table 4.

**Table 4:** Measurements of mediating variables.

Measurement Items	Topics
**User viscosity**	I spend more time on digital music platforms than I do listening to music any other way
	I plan to spend more time on digital music platforms
	If I want to listen to music, I think of digital music first
	I visit digital music platforms as often as possible
**Psychological requirement**	Using digital music platforms, I was able to operate and control the process smoothly
	I am often focused on digital music platforms and feel that time flies
	I’m generally comfortable using digital music platforms
	Paying for digital music is much easier and faster than buying physical albums
	Using digital music platforms can help you discover new artists/songs and have novel experiences
	Paid music gives me more sensory pleasure and enjoyment than free music
	I have experienced a lot of pleasure from using digital music platforms

After completing the questionnaire design, SPSS and AMOS were used to input, process and analyze the collected data [14]. The following 6 main data analysis programs were adopted in this study. Firstly, SPSS software was used for reliability analysis, validity analysis and exploratory factor analysis (EFA) for the pilot test and sample data quality [15]. Then AMOS model was used for confirmatory factor analysis (CFA) and structural equation model (SEM), and the research hypothesis was tested with bootstrap estimation.

Since the measurement items in this study were in the form of Likert 5 scale, Cronbach’s Alpha was used to measure the intrinsic reliability of the questionnaire. The larger the coefficient in Cronbach’s Alpha measurement, the higher the internal consistency of the questionnaire [16]. According to the researchers, if Cronbach’s Alpha coefficient is higher than 0.8, it indicates high reliability, and if Cronbach’s alpha coefficient is between 0.7 and 0.8, it indicates good reliability, and if the value is between 0.6 and 0.7, it indicates that the reliability is acceptable, and if the value is less than 0.6, it indicates poor reliability. Content validity is an objective evaluation index, which refers to whether the setting of each item is representative and comprehensive, whether it is within the research field, and whether it achieves the research purpose Item-Objective Congruence (IOC) is a procedure used to assess the content validity of a project during the development phase of the test development phase and is limited to the evaluation of one-dimensional items or the measurement of specified items. During this process, the questionnaire is checked by an expert in the field of study. The IOC value is calculated based on the score given by each expert. When the IOC value is greater than 0.5, the measured items in the questionnaire can be accepted, indicating that the content validity of the questionnaire is good.

Convergence validity, also known as aggregation validity or internal consistency, is an important indicator when evaluating the quality of a measurement tool or questionnaire. It primarily measures the degree to which different measurement items (such as different questions in a questionnaire) are related to each other under the same concept or construct. When there is a high degree of correlation between the measurement items under the same construct, the convergence validity is good. The mean extraction variance is also an important index for evaluating convergence validity. Its formula is as follows:


$$AVE=\left(\sum^{\lambda^{2}}\right)\;\;╱\;\;\text{n}\;$$

When AVE value is greater than 0.5, it means that the variance of the measured variable explained by its potential variable is greater than the variance explained by its measured error, indicating that the questionnaire has good convergence validity. Discriminant validity (also known as discriminant validity) refers to the degree of decorrelation between a concept and other different concepts, that is, when measuring different variables using different methods, the observed values should be able to distinguish between them. According to existing research [17], in confirmatory factor analysis (CFA), the evaluation method for discriminant validity is to use the square root of AVE and the correlation coefficient between latent variables and other latent variables. If the former is much larger than the latter, then each latent variable shares more variance with its own measurement items than with other measurement items; Further demonstrate that the differences between latent variable measurement items have significant discriminant validity.

This paper carries out exploratory factor analysis (EFA) on the pilot test data, and conducts comprehensive analysis on KMO value, common degree, variance explanation rate value, factor load coefficient value and other indicators to verify the validity level of the data. The data in this study was rotated using the maximum variance rotation method (varimax) in order to find out the corresponding relationship between the factor and the research item [18]. KMO value was used to judge the suitability of information extraction, common degree value was used to exclude unreasonable research items, variance interpretation rate value was used to illustrate the level of information extraction, and factor load coefficient was used to measure the correspondence between factors (dimensions) and items. Combined with the factor load coefficient, confirm whether the correspondence between the factor (dimension) and the research item is consistent with the expectation. If it is consistent, it indicates the validity; otherwise, it needs to be re-adjusted. When the absolute value of factor load coefficient is greater than 0.4 (0.3 standard is also adopted in foreign countries), it indicates that there is a corresponding relationship between the option and the factor.

SEM is a statistical method of analyzing relationships between variables that can be used to build global models and estimate and test causal relationships. It can not only handle multiple dependent variables at the same time, but also allow them and the independent variables to have a certain measurement error, while estimating factor structures and relationships, allowing for a more elastic measurement model, and estimating the degree of fit of the model. Due to the existence of variable relationships in this study and the interpretation of the AMOS results’ View Text ‘, we gradually expanded the initial model by adjusting the SEM correction index (M.I.) to correct the AMOS.

In this paper, AMOS software is used to test the structural equation model, the non-parametric percentile bootstrapping method is used to correct the deviation, and the percentile 95% confidence interval is set in AMOS software (24–28). The confidence interval is the possible range of the whole parameter. A 95% confidence interval is when the overall parameter is within this range with about 95% probability, or when the overall parameter is within this range but has only 95% confidence. If the upper and lower values of the confidence interval have different signs, that is, the range between the upper and lower values contains 0, then the coefficient of the confidence interval is not significant; If both values of the confidence interval are positive, then the range between them does not contain 0, indicating that the coefficient of the confidence interval is significant. Therefore, Bootstrapping estimation method combined with structural equation model (SEM) was selected to verify the research hypothesis. In the previous chapters, a 4-step research design procedure is developed, and the quantitative research is conducted according to it. (1) From the measurement dimension, we designed operational question items for all variables and formed a questionnaire with 42 question items. (2) Based on a combination of purposive sampling and stratified sampling, 431 samples were obtained. (3) The data analysis procedure is summarized in detail. (4) Based on this, the validity analysis (content validity - IOC test and structure validity - EFA) and reliability analysis were carried out for 36 variable data.

## 3. Results and discussion

In this section we discuss the results of the survey. We begin by describing the respondents to our survey. Then, descriptive statistics and analysis of the data variables are conducted (with indicators such as frequency, percentage, mean, and standard deviation), and the quality of the sample data is evaluated using reliability, validity analysis, and confirmatory factor analysis (CFA) (with analysis indicators such as factor loading, composite reliability (CR), average variance extracted (AVE), and model fit indices). Finally, path analysis and Bias-Corrected Bootstrap procedures are used to test the research hypotheses. The data in the Demographic Profile Questionnaire (Part1) of the respondents were analyzed and expressed in frequency and percentage terms, including gender, age, average monthly consumption, sources of living expenses, monthly expenses for digital music, living expenses, spending methods, etc. Through data analysis, the statistical results are shown in Table 5 There were 221 males (51.28%) and 210 females (48.72%) in the surveyed sample, with a slightly higher proportion of males than females. In grade level, 420 sophomores (97.45 percent) were enrolled, while students from other grades accounted for a relatively small proportion. In terms of monthly consumption, 226 students (52.44 percent) spend between 1,000 and 1,500 yuan, and 134 students (31.09 percent) spend between 1,500 and 2,000 yuan. In terms of the source of living expenses, 318 students (73.78 percent) came from their families, followed by 112 students (25.99 percent) who came partly from their families and partly earned by themselves. In terms of monthly expenses for digital music, the largest number of students were between 1 and 200, with 236 (54.76%), followed by no consumption, with 192 (44.55%). There are 234 people (54.29%) who think that their living expenses are just enough, followed by 146 people (33.87%) who think that they have enough. In terms of the way they spend money, 275 people (63.81 percent) said they plan to spend while they can, followed by 71 people (16.47 percent) who want to save money.

**Table 5:** Frequency analysis of the sample.

Name	Options	Frequency	Percentage (%)	Cumulative percentage (%)
Gender	male	221	51.28	51.28
	female	210	48.72	100
Grade	Freshman Year	5	1.16	1.16
	Sophomore year	420	97.45	98.61
	Juniors	4	0.93	99.54
	Senior	2	0.46	100
Average monthly spending during the school year	600-1000.	38	8.82	8.82
	1001-1500.	226	52.44	61.25
	1501-2000.	134	31.09	92.34
	Over 2000	33	7.66	100
Source of living expenses	All from family	318	73.78	73.78
	Partly from family, partly earned	112	25.99	99.77
	Earn it all by yourself	1	0.23	100
Monthly expenses for digital music	There is no	192	44.55	44.55
	1-200.	236	54.76	99.3
	201-500.	1	0.23	99.54
	500 +	2	0.46	100
You feel your living expenses	To have enough	146	33.87	33.87
	Just enough	234	54.29	88.17
	Not enough	51	11.83	100
How to spend money	Plan it all before you spend it	42	9.74	9.74
	Save if you can	71	16.47	26.22
	Spend as much as you want	35	8.12	34.34
	Plan as you spend	275	63.81	98.14
	Other	8	1.86	100
Total		431	100	100

The demographic profile of the respondents in this study is diverse in terms of study courses and economic backgrounds. However, we acknowledge that the grade distribution is skewed, with 97.45% being second-year students. This imbalance may reduce the representativeness of the entire student population. Future studies could adopt stratified random sampling across all grades to enhance generalizability. Despite this limitation, the data quality was rigorously ensured through validity and reliability tests (Cronbach’s α > 0.8).The observed variables of the study are examined in the second part of the questionnaire. In this paper, descriptive statistical analysis is conducted on all variables from two levels.

The first level is descriptive statistical analysis for each item. Mean and standard deviation are included in the analysis indicators. The statistical results are shown in Table 6. According to the previous assumptions, the study selected perceived value, behavioral attitude, subjective norms and user participation as independent variables of this study, and measured them with relevant scales. Among them, the mean value of perceived value is between 3.657–4.049, which indicates that the perceived value of college students surveyed by the research is above the medium level. The mean value of behavioral attitude questionnaire is between 3.51 and 3.893, which indicates that the behavioral attitude of college students investigated by the research are at the upper middle level. The mean value of the subjective norm questionnaire was between 3.564 and 3.893, indicating that the subjective norm of college students surveyed by the research was at the upper middle level. The mean of user participation was between 3.418 and 3.696, indicating that the user participation of college students surveyed by the research was at the upper middle level.

**Table 6:** Descriptive analysis of observed variables.

Variables	Items	Sample size	Average	Standard Deviation
Perceived value	PV6	431	4.032	1.002
Behavior attitude	BA4	431	3.893	1.008
Subjective Norms	SN2	431	4.086	1.027
User engagement	UP4	431	3.696	1.146
Stickiness	UV4	431	4.176	0.948
Psychological need	PR4	431	4.132	0.985
Willingness to spend	CW2	431	3.645	1.121

The dependent variable of this study is consumption intention, and the results show that the average intention to consume of the university students is between 3.55 and 3.645, which indicates that the level of consumption intention of college students is good. User stickiness and psychological needs are selected as the mediating variables. It shows that the overall mean of the questionnaire on user stickiness of college students is 4.048, and the mean of its sub-dimensions is between 3.981–4.176, which indicates that the stickiness of college students is above the medium level. The overall mean of the survey data on psychological needs of college students is 3.941, and the mean of its sub-dimensions is between 3.696–4.035, which indicates that the psychological needs of college students are at the upper middle level.

The second level is an overall descriptive statistical analysis of all the variables, the results of which are presented in Table 7. It is not difficult to see from the results that the average values of perceived value, behavioral attitude, subjective norms, user participation, user stickiness, psychological needs, and consumption intention are 3.858, 3.704, 4.071, 3.581, 4.048, 3.941, and 3.599, respectively. It is obvious that all these values exceed 3, indicating that the measurement results of all observed variables are good.

**Table 7:** Overall descriptive statistics of all observed variables.

Variables	Sample Size	Average	Standard Deviation
Perceived value	431	3.858	0.852
Behavioral attitude	431	3.704	0.842
Subjective norm	431	4.071	0.847
User participation	431	3.581	0.988
User stickiness	431	4.048	0.791
Psychological demand	431	3.941	0.791
Consumption intention	431	3.599	0.981

Based on the theory of correlation analysis, it was found that there is a correlation between UV, PR, CW and PV, BA, SN, UP, respectively, and they were studied. Pearson correlation coefficient can be used to indicate the strength of the correlation relationship. The specific analysis results are shown in Table 8.

**Table 8:** Correlation analysis.

	PV	BA	SN	UP	UV	PR	CW
PV	1						
BA	0.293 * * *	1					
SN	0.365 * * *	0.320 * * *	1				
UP	0.287 * * *	0.331 * * *	0.340 * * *	1			
UV	0.313 * * *	0.330 * * *	0.329 * * *	0.380 * * *	1		
PR	0.344 * * *	0.401 * * *	0.439 * * *	0.338 * * *	0.320 * * *	1	
CW	0.443 * * *	0.460 * * *	0.487 * * *	0.498 * * *	0.453 * * *	0.491 * * *	1

* *p* < 0.05 ** *p* < 0.01 *** *p* < 0.001.

The results showed that UV was significantly associated with PV, BA, SN, and UP. The correlation coefficient values between them are 0.313, 0.330, 0.329, and 0.380, respectively. These correlation coefficient values are all greater than 0, indicating a positive correlation between UV and the four terms PV, BA, SN, and UP.

The results showed that PR was significantly associated with PV, BA, SN, and UP. The correlation coefficient values between them are 0.344, 0.401, 0.439, and 0.338, respectively. These correlation coefficient values are all greater than 0, indicating a positive correlation between PR and the four terms PV, BA, SN, and UP.

Similarly, significant differences were observed between CW and PV, BA, SN, and UP. The correlation coefficient values between them are 0.443, 0.460, 0.487, and 0.498, respectively. These correlation coefficient values are all greater than 0, indicating a positive correlation between CW and the four terms PV, BA, SN, and UP.

In addition, the correlation coefficient between CW and UV is 0.453 and shows significance at the 0.01 level, indicating a significant positive correlation between CW and UV. The correlation coefficient between CW and PR is 0.491 and shows a significance level of 0.01, indicating a significant positive correlation between CW and PR.

The validity evaluation of sample data includes two aspects: content validity evaluation and structural validity evaluation. Before conducting exploratory factor analysis (EFA), KMO and Bartlett tests should be performed first.

Upon analyzing the results, it was found that the KMO value of the survey questionnaire was 0.933, which is clearly greater than 0.7. The results also showed that the chi-square value of Bartlett’s sphericity test was 8106.028, the degree of freedom was 630, and the significance was 0.000. The data can be effectively extracted, indicating that there are common structural factors among the variables in the questionnaire, which can be analyzed for factors as shown in Table 9.

**Table 9:** KMO and Bartlett’s test results.

KMO value		0.933
Bartlett’s sphericity test	Approximate Chi-square	8106.028
	d*f*	630
	*p* value	0.000

**Fig 2:**
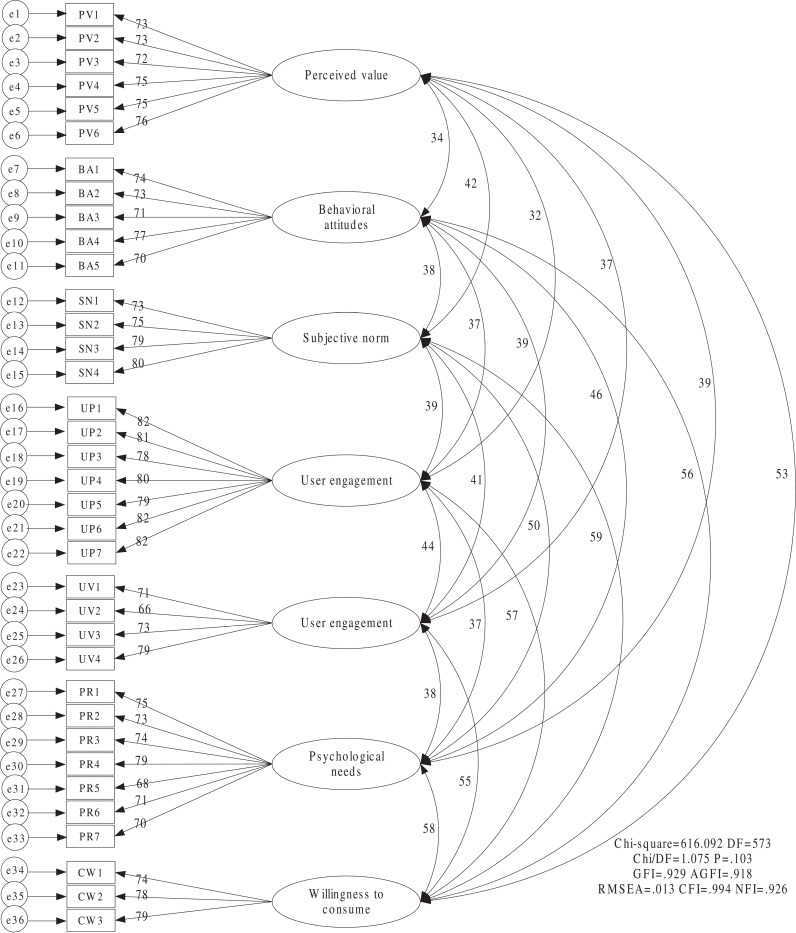
Confirmatory factor Analysis diagram.

Through the reliability and validity analysis of this section, it was found that the evaluation of sample data has high reliability and validity. AMOS software was used to analyze the measurement model composed of independent variables by fixed loading method and maximum likelihood method (ML). The model consists of 7 variables, such as independent variable perceived value, behavioral attitude, subjective norm, user participation, dependent variable consumption intention and intermediate variable user stickiness and psychological demand. Based on the confirmatory factor analysis (CFA) model, analyze independent variables, mediating variables, and dependent variables. It can be seen that this CFA model involves 7 factors and 36 measurement indicators as shown in Fig 2.

According to the T rule of the measurement model identification criteria (T≤P(P+1)/2)), the CFA model has a total of 36 observation indicators, so the total degree of freedom isq(q+1)/(2=666), and a total of 79 parameters need to be estimated:36 factor loads, error variances of 36 measurement indicators and correlation coefficients between 7 factors. T=79<666, which meets the necessary conditions for model identification [19].

On the premise that the data in the model meets the standards, the impact paths between variables are determined based on regression analysis, and the mediating effects of the mediating variables are tested using Bootstrap sampling method.

Table 10 shows that perceived value, behavioral attitude, subjective norms and user participation, as independent variables, all have significant positive effects on user stickability and psychological demand of mediating variables. Specifically, when studying the impact of perceived value on user stickiness, the standardized path coefficient value was 0.147 > 0 and showed a significance level of 0.05 (CR = 2.497, p = 0.013 < 0.05), indicating that perceived value has a significant positive effect on user stickiness. Similarly, when studying the impact of behavioral attitudes on user stickiness, 0.184 > 0, CR = 3.072, p = 0.002 < 0.01; When studying the impact of subjective norms on user stickiness, 0.183 > 0, CR = 2.914, p = 0.004 < 0.01; When studying the impact of user participation on user stickiness, 0.254 > 0, CR = 4.401, p < 0.001。These indicate that behavioral attitudes, subjective norms, and user participation have a significant positive impact on user stickiness.

**Table 10:** Statistical data of path analysis among variables.

	Path		Non-standardized path coefficients	S.E.	C.R.	P	Standardized path coefficient
Stickiness	←	Perceived value	0.125	0.05	2.497	0.013	0.147
Stickiness	←	Behavioral attitude	0.162	0.053	3.072	0.002	0.184
User engagement	←	Subjective norm	0.17	0.058	2.914	0.004	0.183
Stickiness	←	User engagement	0.183	0.042	4.401	* * *	0.254
Psychological needs	←	Perceived value	0.133	0.051	2.605	0.009	0.142
Psychological needs	←	Behavioral attitude	0.253	0.055	4.596	* * *	0.26
Psychological needs	←	Subjective norm	0.312	0.062	5.056	* * *	0.304
Psychological needs	←	User engagement	0.092	0.041	2.221	0.026	0.116
Willingness to spend	←	Perceived value	0.186	0.054	3.433	* * *	0.173
Willingness to spend	←	Behavioral attitude	0.217	0.06	3.644	* * *	0.194
Willingness to spend	←	Subjective norm	0.233	0.066	3.51	* * *	0.198
Willingness to spend	←	User engagement	0.209	0.045	4.59	* * *	0.228
Willingness to spend	←	Stickiness	0.208	0.069	3.016	0.003	0.164
Willingness to spend	←	Psychological needs	0.203	0.063	3.209	0.001	0.177
PV1	←	Perceived value	1				0.726
PV2	←	Perceived value	0.948	0.067	14.202	* * *	0.726
PV3	←	Perceived value	0.972	0.069	14.11	* * *	0.721
PV4	←	Perceived value	0.946	0.065	14.652	* * *	0.749
PV5	←	Perceived value	1.041	0.071	14.592	* * *	0.746
PV6	←	Perceived value	0.926	0.062	14.82	* * *	0.758
BA1	←	Behavioral attitude	1				0.735
BA2	←	Behavioral attitude	0.985	0.07	14.071	* * *	0.729
BA3	←	Behavioral attitude	0.943	0.068	13.769	* * *	0.713
BA4	←	Behavioral attitude	0.982	0.066	14.825	* * *	0.771
BA5	←	Behavioral attitude	0.993	0.074	13.507	* * *	0.699
SN1	←	Subjective norm	1				0.728
SN2	←	Subjective norm	1.026	0.071	14.378	* * *	0.75
SN3	←	Subjective norm	1.123	0.075	15.077	* * *	0.79
SN4	←	Subjective norm	1.014	0.067	15.165	* * *	0.796
UP1	←	User engagement	1				0.816
UP2	←	User engagement	0.965	0.049	19.616	* * *	0.814
UP3	←	User engagement	0.959	0.052	18.431	* * *	0.779
UP4	←	User engagement	0.945	0.049	19.149	* * *	0.8
UP5	←	User engagement	0.984	0.053	18.65	* * *	0.785
UP6	←	User engagement	1.007	0.051	19.761	* * *	0.818
UP7	←	User engagement	1.009	0.05	19.981	* * *	0.824
UV1	←	Stickiness	1				0.714
UV2	←	Stickiness	0.953	0.079	12.09	* * *	0.659
UV3	←	Stickiness	1.06	0.08	13.211	* * *	0.73
UV4	←	Stickiness	1.077	0.077	14.008	* * *	0.793
PR1	←	Psychological needs	1				0.747
PR2	←	Psychological needs	0.967	0.065	14.903	* * *	0.734
PR3	←	Psychological need	1.011	0.067	15.052	* * *	0.741
PR4	←	Psychological needs	0.93	0.063	14.76	* * *	0.727
PR5	←	Psychological need	0.979	0.071	13.721	* * *	0.678
PR6	←	Psychological needs	0.936	0.065	14.346	* * *	0.708
PR7	←	Psychological need	0.928	0.065	14.258	* * *	0.704
CW1	←	Willingness to spend	1				0.744
CW2	←	Willingness to spend	0.982	0.066	14.934	* * *	0.776
CW3	←	Willingness to spend	1.011	0.067	15.091	* * *	0.786

When studying the impact of perceived value on psychological needs, the standardized path coefficient value is 0.142 > 0, and this path shows a significance level of 0.05 (CR = 2.605, p = 0.009 < 0.05). This indicates that perceived value has a significant positive impact on psychological needs. Similarly, when studying the impact of behavioral attitudes on psychological needs, 0.26 > 0, CR = 4.596, p=<0.001; When studying the impact of subjective norms on psychological needs, 0.304 > 0, CR = 5.056, p=<0.001; When studying the impact of user participation on psychological needs, 0.116 > 0, CR = 2.221, p < 0.001。 These indicate that behavioral attitudes, subjective norms, and user participation have a significant positive impact on psychological needs.

The independent variables (perceived value, behavioral attitude, subjective norms, user participation, and mediating variables such as user stickiness and psychological needs) all have a significant positive impact on the dependent variable (willingness to consume). Specifically, when studying the impact of perceived value on consumption intention, the standardized path coefficient value was 0.173 > 0, and this path showed a significance level of 0.05 (CR = 3.433, p=<0.001), indicating that perceived value has a significant positive impact on consumption intention. Similarly, when studying the impact of behavioral attitudes on consumer willingness, 0.198 > 0, CR = 3.51, p=<0.001; When studying the impact of user participation on consumption intention, 0.164 > 0, CR = 3.016, p = 0.003 < 0.01; When studying the impact of psychological needs on consumer willingness, 0.177 > 0, CR = 3.209, p = 0.001 < 0.011。 These indicate that behavioral attitudes, user participation, and psychological needs have a significant positive impact on consumer willingness.

Table 11 shows that all indexes in the path analysis model constructed by the research reach the standard level, indicating that the model has a good fit.

**Table 11:** Model fitting index.

Common Indicators	χ^2^	d*f*	*p*	χ^2^/d*f*	RMSEA	GFI	AGFI	CFI	NFI	TLI	IFI
Judging criteria	–	–	> 0.05	< 3	< 0.10	> 0.9	> 0.9	> 0.9	> 0.9	> 0.9	> 0.9
Value	617.992	574	0.099	1.077	0.013	0.929	0.918	0.994	0.926	0.994	0.994

In order to study the mediation effect, based on the suggestion of Preacher and Hayes, 5000 times bootstrap repeated sampling method is adopted to deal with the situation that the data in this study do not conform to the normal distribution. And select 95% confidence interval of percentile and 95% confidence interval of Bias-Corrected to test the significance of path coefficients. Therefore, we examined the Critical Ratio (C.R.), i.e., lower bound and upper bound, and two-tailed significance to test whether the effect between potential variables is significant.

The results of Bootstrapping estimates are shown in Table 12.

**Table 12:** Results of Bootstrapping analysis.

Paths	Effect size	Lower	Upper	P	Effect proportion
Intermediate effect					
PV_UV_CW	0.026	0.004	0.069	0.019	10.88%
BA_UV_CW	0.034	0.007	0.084	0.006	11.26%
SN_UV_CW	0.035	0.006	0.096	0.013	10.57%
UP_UV_CW	0.038	0.012	0.083	0.002	14.29%
PV_PR_CW	0.027	0.005	0.072	0.011	11.30%
BA_PR_CW	0.051	0.016	0.112	0.004	16.89%
SN_PR_CW	0.063	0.023	0.124	0.004	19.03%
UP_PR_CW	0.019	0.001	0.051	0.038	7.14%
Direct effects					
PV-CW	0.186	0.058	0.307	0.006	
BA-CW	0.217	0.098	0.344	0.002	
SN-CW	0.233	0.096	0.391	0.001	
UP-CW	0.209	0.106	0.304	0.001	

Note: Lower refers to the lower limit of the 95% interval of the Bootstrap sample, and Upper refers to the upper limit of the 95% interval of the Bootstrap sample.

The results in Table 4.16 show that in the path of “PV=>UV=>CW”, the indirect effect of “UV” has 95%CI confidence interval (0.004, 0.069), the confidence interval does not include 0, and the significance P = 0.019 < 0.05, indicating that the intermediary effect is statistically significant and the intermediary effect exists. The effect size (non-standard) was 0.026, and the proportion of intermediate effect size was 10.88%.

In the path of “BA=>UV=>CW”, the indirect effect of “BA” has 95%CI confidence interval (0.007, 0.084), the confidence interval does not include 0, and the significance P = 0.006 < 0.05, indicating that the intermediary effect is statistically significant and the intermediary effect exists. The effect size (non-standard) was 0.034, and the proportion of mediating effect size was 11.26%. In the path of “SN=>UV=>CW”, 95%CI confidence interval (0.006, 0.096) for indirect effect of “SN”, confidence interval excluding 0, significance P = 0.013 < 0.05, indicating that the intermediary effect is statistically significant and the intermediary effect exists. The effect size (non-standard) was 0.035, and the proportion of intermediate effect size was 10.57%. In the path of “UP=>UV=>CW”, the indirect effect of “UP” was 95%CI confidence interval (0.012, 0.083), the confidence interval did not include 0, and the significance P = 0.002 < 0.05, indicating that the intermediary effect was statistically significant and the intermediary effect existed. The effect size (non-standard) was 0.038, and the proportion of mediating effect size was 14.29%.

In the path of “PV=>PR=>CW”, the indirect effect of “PV” has 95%CI confidence interval (0.005, 0.072), the confidence interval does not include 0, and the significance P = 0.011 < 0.05, indicating that the intermediary effect is statistically significant and the intermediary effect exists. The effect value (non-standard) was 0.027, and the proportion of the intermediary effect size was 11.30%.

In the path of “BA=>PR=>CW”, the indirect effect of “BA” has 95%CI confidence interval (0.016, 0.112), the confidence interval does not include 0, and the significance P = 0.004 < 0.05, indicating that the intermediary effect is statistically significant and the intermediary effect exists. The effect size (non-standard) was 0.051, and the proportion of mediating effect size was 16.89%. In the path of “SN=>PR=>CW”, the indirect effect of “BA” had 95%CI confidence interval (0.016, 0.112), the confidence interval did not include 0, and the significance P = 0.004 < 0.05, indicating that the intermediary effect was statistically significant and the intermediary effect existed. The effect size (non-standard) was 0.051, and the proportion of mediating effect size was 16.89%.

In the path of “UP=>PR=>CW”, the indirect effect of “UP” was 95%CI confidence interval (0.001, 0.051), the confidence interval did not include 0, and the significance P = 0.038 < 0.05, indicating that the intermediary effect was statistically significant and the intermediary effect existed. The effect size (non-standard) was 0.019, and the proportion of mediating effect size was 7.14%.

Total effect size of PV-CW =0.186+0.026+0.027=0.239

Total effect size for BA-CW =0.217+0.034+0.051=0.302

Total effect size for SN-CW =0.233+0.035+0.063=0.331

Total effect size for UP-CW =0.209+0.038+0.019=0.266

**Fig 3:**
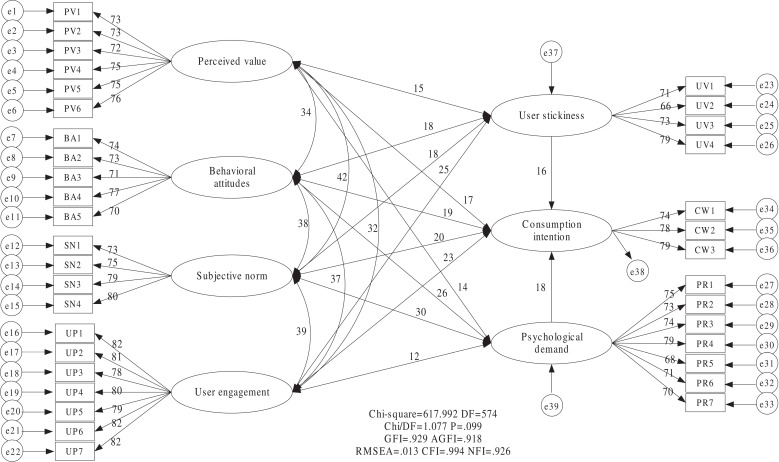
Test results for conceptual framework annotations.

Therefore, overall, subjective norms have the greatest influence on consumption intention, while perceived value has the least influence on consumption intention. Based on the results of bootstrapping estimates, we show the results of the conceptual framework in Fig 3.

Through empirical analysis of structural equation modeling (SEM) with bootstrap estimation, the test results of four research hypotheses were obtained, summarized in Table 13. Table 13 shows that all four hypotheses are supported, which indicates that our validation analysis is scientifically sound. In addition, compared with regression coefficient, subjective norms have the greatest influence on consumption intention, while perceived value has the least influence on consumption intention.

**Table 13:** Test results of four research hypotheses.

Hypotheses	Test results
H1: Perceived value positively affects college students’ consumption intention	True
H2: Behavior attitude will positively affect college students’ consumption intention	True
H3: Subjective normative justice will affect college students’ willingness to consume	True
H4: The positive effect of consumer participation on college students’ consumption intention	True

To gain a more comprehensive understanding of the implications of the findings in this study, we compared them with relevant international research. Existing research [20,21] has explored the impact of online music videos on recorded music sales, finding that music videos can effectively boost record sales. This echoes our study’s results, where factors such as user engagement and behavioral attitudes have significant effects on college students’ willingness to consume digital music. Despite differences in research subjects and market environments, these commonalities suggest that digital music platforms should focus on enhancing user experience and a sense of participation when formulating marketing strategies. Furthermore, when investigating the role of popular music in cultural identity formation in the streaming era, the importance of music as a tool for shaping cultural identity was emphasized [22–26]. This perspective is also reflected in our study, as college students may be influenced by subjective norms (such as recommendations from friends and family) when choosing to consume digital music. Therefore, digital music platforms can leverage word-of-mouth marketing through channels such as social media to expand their user base [27–30]. Based on the findings of this study, specific marketing strategies can be formulated for digital music platforms and the music industry.

User experience enhancement: By optimizing user interfaces, providing high-quality music content, and offering personalized recommendation systems, the perceived value of digital music platforms among users can be improved. This, in turn, helps to increase user retention and, consequently, boost consumption intentions [31–35]. User engagement encouragement: Online music events and user creation contests can be organized to encourage active participation in the construction and interaction of digital music platforms. This not only increases user involvement but also attracts more new users through word-of-mouth promotion. Social media marketing utilization: Social media platforms (such as Weibo and WeChat) can be leveraged for word-of-mouth marketing, expanding brand influence through user shares, comments, and other interactions. Additionally, influencers, musicians, and other figures can be invited for collaborative promotions to attract more targeted users [36–39]. Differentiated marketing strategy formulation: Differentiated marketing strategies should be developed for college student groups based on factors such as gender, grade, and consumption level. For example, high-end membership services can be offered to students with higher consumption levels, while discounted packages or free trials can be provided to those with lower consumption levels [40].

## 4. Conclusion and limitation

### 4.1. Conclusion

Using a combination of purposeful sampling and stratified sampling, and employing AMOS software for bootstrap estimation of structural equation modeling (SEM), this study conducted a quantitative investigation into the digital music consumption of college students in the Chengdu area of China (with a sample size of 431 college students). The study examines how perceived value, behavioral attitude, subjective norms, user stickiness, and psychological needs collectively influence their consumption intentions. These factors interact with and promote each other, together forming an important system of factors affecting college students’ consumption decisions. It was found that independent variables (perceived value, behavioral attitude, subjective norms, user participation) have a significant positive impact on the dependent variable (willingness to consume). The perceived value of independent variables, behavioral attitudes, subjective norms and user participation have significant positive effects on user stickiness and psychological needs of intermediary variables. User stickiness and psychological needs play a significant mediating role in perceived value, behavioral attitude, subjective norms, and user participation on consumption intention. Our findings align with prior studies that highlight the importance of psychological needs in digital consumption. However, the dominance of second-year students in our sample may limit direct comparability with studies involving multi-grade cohorts. Future research should validate these results across diverse academic stages to strengthen generalizability.

### 4.2. Limitations and future research directions

While this study provides valuable insights into the factors influencing college students’ willingness to consume digital music, several limitations should be acknowledged. In addition to TAM and TPB, constructs related to status, personality traits, and ‘generational’ affiliation may also be relevant to the consumption of intangible services closely linked to youth. Future research could incorporate these insights to further validate the theoretical model. Chengdu was chosen due to its vibrant youth culture, significant student population, and active promotion as a music industry hub, making it representative of China’s digital music trends. However, we acknowledge limitations in generalizing findings to other regions or international contexts. Regional differences in economic development, cultural preferences, and digital infrastructure may influence consumption patterns. For broader applicability, future studies should include diverse regions across China and replicate the research in international markets to account for cultural and market variations. While Chengdu provides valuable insights, expanding the scope will enhance the generalizability of our findings. Additionally, the cross-sectional design captures static behavioral intentions but does not account for dynamic changes in consumption patterns over time. The study did not differentiate between consumption preferences for specific music genres (e.g., pop, classical, hip-hop), which may exhibit distinct behavioral patterns. Future research could incorporate genre-specific analyses to refine marketing strategies.

Additionally, emerging technologies such as artificial intelligence (AI) and blockchain are reshaping digital music ecosystems. AI-driven recommendation algorithms could enhance personalization by aligning with users’ psychological needs and behavioral attitudes, while blockchain technology may address copyright challenges and incentivize user participation through transparent royalty distribution. However, these technological impacts were beyond the scope of this study. Future investigations should explore how such innovations interact with traditional consumption factors.

### 4.3. Strategic implications for digital music platforms

These results provide practical guidance for digital music platforms, helping them effectively improve users’ consumption tendencies by enhancing perceived value, increasing user participation, and meeting psychological needs. Platforms can leverage social influence and personalized recommendations to cultivate user loyalty and strengthen subjective norms, thereby promoting long-term consumption behavior. Additionally, the research supports platform optimization of product design and services, as well as the development of precise marketing strategies to enhance market competitiveness and user satisfaction. To operationalize the findings, digital music platforms should adopt a multi-pronged strategy that integrates AI-powered features to enhance engagement, particularly on emerging platforms like TikTok Music, where short-form video content and music discovery are intertwined. Addressing psychological needs can be achieved by designing tiered subscription models that cater to budget constraints while offering exclusive content to amplify perceived value. Collaboration with blockchain networks can ensure fair compensation for artists, aligning with college students’ ethical consumption attitudes. Furthermore, platforms should tailor marketing efforts to dominant genres among youth and leverage TikTok’s viral trends to amplify reach and engagement.

## Supporting information

S1 Appendix Survey questionnaire questions. (DOCX)

S2 Appendix Survey questionnaire statistics. (PDF)
