# Supplementary material for: A statistical analysis of causal factors influencing college student’s willingness to consume digital music
Source: PLoS One. 2025 Jun 2;20(6):e0324168. doi: 10.1371/journal.pone.0324168 (PMC12129152; doi:10.1371/journal.pone.0324168)
Supplement: S1 Appendix — (DOCX) [file pone.0324168.s001.docx]

**Appendix A. Supplemental Materials**

Table S1 Strongly Disagree 2 Disagree 3 Neutral 4 Agree 5 Strongly Agree

| Symbol | Dimensions | Question number | Title |
| --- | --- | --- | --- |
| PV1 | Perceived value | 1 | In my opinion, the paid music business is worth buying and cost-effective |
| PV2 |  | 2 | I think the paid music business makes it easier and more convenient to listen to and download music |
| PV3 |  | 3 | Paid music allows me to enjoy more music resources |
| PV4 |  | 4 | Paid music allows me to enjoy a better audio-visual experience |
| PV5 |  | 5 | Paying for music can get me more membership benefits |
| PV6 |  | 6 | Compared with switching apps to listen to songs, buying paid music directly is more convenient, quality and cost-effective |
| BA1 | Behavior Attitude | 7 | I think digital music charges are a result of complying with copyright protection laws |
| BA2 |  | 8 | I think it is ethical to charge for digital music as a commodity |
| BA3 |  | 9 | Enjoying free music is a user habit cultivated by the Internet that is hard to change |
| BA4 |  | 10 | It is difficult to strengthen users' awareness of music copyright and cultivate paid music consumption habits |
| BA5 |  | 11 | I think charging for digital music will encourage musicians to produce better work |
| SN1 | Subjective norms | 12 | More people around me have been understanding of my move to buy digital music |
| SN2 |  | 13 | More people around support the idea of charging for digital music |
| SN3 |  | 14 | More people around have purchased paid music business behavior (single album membership online concert) |
| SN4 |  | 15 | If my friends and family recommend me to buy paid music, I try to buy it |
| UP1 | User engagement | 16 | I create different playlists for different types of music |
| UP2 |  | 17 | I follow and bookmark certain artists or music |
| UP3 |  | 18 | I like certain music when I listen to digital music |
| UP4 |  | 19 | I will participate in reviews while listening to music on digital music platforms |
| UP5 |  | 20 | I will interact and communicate with other users in the digital music related community |
| UP6 |  | 21 | I will share and forward some digital music to others |
| UP7 |  | 22 | I will post and repost information about digital music or singers on social networks or other platforms to communicate with others. |
| UV1 | User stickiness | 23 | I would stay on digital music platforms longer than I would listen to music any other way |
| UV2 |  | 24 | I plan to extend my stay on digital music platforms |
| UV3 |  | 25 | If I want to listen to music, I prioritize digital music platforms |
| UV4 |  | 26 | I will visit digital music platforms as often as possible |
| PR1 | Psychological needs | 27 | When using the digital music platform, I can operate and control the process smoothly |
| PR2 |  | 28 | I'm always focused on digital music platforms and feel like time flies |
| PR3 |  | 29 | I feel comfortable using digital music platforms overall |
| PR4 |  | 30 | Buying paid digital music is easier and faster than buying physical albums |
| PR5 |  | 31 | Digital music platform use helps to discover new singers/songs and have novel experiences |
| PR6 |  | 32 | Paid music gives me more sensual pleasure and enjoyment than free music |
| PR7 |  | 33 | I have experienced a lot of pleasure in using digital music platforms |
| CW1 | Willingness to consume | 34 | If I come across paid music that I like, I try to buy it |
| CW2 |  | 35 | I can see myself buying paid music in the future |
| CW3 |  | 36 | I would love to recommend good paid music to friends and family |

1 Your gender

A Male and B female

2 Your grade

A Freshman B Sophomore C Junior D Senior

3 Average monthly spending while in school(¥)

A 600-1000 B 1001-1500 C 1501-2000 D 2000 or more

4 Monthly expenses for digital music

A 0 ¥ B 0-100¥ C 100-200¥ D 200¥ and above

5 How long you listen to music on an average day

A less than 30 minutes B 30 minutes -1 hour C 1-2 hours D 2 hours or more

6 What is your preferred consumption behavior on the music platform? (Multiple choice)

A Member B Digital album or single C Music Streaming Package D Music peripherals E Other
