# Supplementary material for: A statistical analysis of causal factors influencing college student’s willingness to consume digital music
Source: PLoS One. 2025 Jun 2;20(6):e0324168. doi: 10.1371/journal.pone.0324168 (PMC12129152; doi:10.1371/journal.pone.0324168)
Supplement: S2 Appendix — (PDF) [file pone.0324168.s002.pdf]

大学生数字音乐消费意愿情况调查问卷

第1题：您的性别 [单选题]

选项	小计	比例
男	221	51.16%
女	211	48.84%
**本题有效填写人次**	**432**	

第2题：年级 [单选题]

选项	小计	比例
大一	5	1.16%
大二	421	97.45%
大三	4	0.93%
大四	2	0.46%
**本题有效填写人次**	**432**	

第3题：在校期间的平均月消费 [单选题]

选项	小计	比例
600-1000	38	8.8%
1000-1500	226	52.31%
1500-2000	134	31.02%
2000以上	34	7.87%
**本题有效填写人次**	**432**	

第4题：生活费来源 [单选题]

选项	小计	比例
全部来自家庭	319	73.84%
部分来自家庭，部分靠自己赚取	112	25.93%
全部靠自己赚取	1	0.23%
**本题有效填写人次**	**432**	

第5题：每月用于数字音乐的费用 [单选题]

选项	小计	比例
无	193	44.68%
1-200	236	54.63%
200-500	1	0.23%

选项	小计	比例
500以上	2	0.46%
**本题有效填写人次**	**432**	

第6题：您觉得自己的生活费 [单选题]

选项	小计	比例
有富余	147	34.03%
刚好够用	234	54.17%
不够	51	11.81%
**本题有效填写人次**	**432**	

第7题：花钱方式是 [单选题]

选项	小计	比例
全部计划好再花	42	9.72%
能省则省	71	16.44%
想花就花	35	8.1%
一边花一边打算	276	63.89%
其他	8	1.85%
**本题有效填写人次**	**432**	

第8题：我会因为对某一数字音乐APP的第一印象而选择是否进行购买 [单选题]

选项	小计	比例
完全不符合	51	11.81%
不符合	72	16.67%
一般	175	40.51%
符合	119	27.55%
完全符合	15	3.47%
**本题有效填写人次**	**432**	

第9题：我会根据他人对某一数字音乐APP的评价而影响其进行第一印象的评价 [单选题]

选项	小计	比例
完全不符合	49	11.34%
不符合	81	18.75%
一般	173	40.05%
符合	123	28.47%

选项	小计	比例
完全符合	6	1.39%
**本题有效填写人次**	**432**	

第10题：我更在乎收费的价格，而不是版权是否能够得到保护 [单选题]

选项	小计	比例
完全不符合	52	12.04%
不符合	106	24.54%
一般	173	40.05%
符合	75	17.36%
完全符合	26	6.02%
**本题有效填写人次**	**432**	

第11题：我更在乎版权是否能够得到保护，而不是收费的价格 [单选题]

选项	小计	比例
完全不符合	30	6.94%
不符合	42	9.72%
一般	220	50.93%
符合	107	24.77%
完全符合	33	7.64%
**本题有效填写人次**	**432**	

第12题：相较于价格，在购买数字音乐时，用户体验是我更加关注的范畴 [单选题]

选项	小计	比例
完全不符合	13	3.01%
不符合	21	4.86%
一般	137	31.71%
符合	201	46.53%
完全符合	60	13.89%
**本题有效填写人次**	**432**	

第13题：我更倾向于购买我参与过活动的品牌好形象好的数字音乐APP [单选题]

选项	小计	比例
完全不符合	13	3.01%
不符合	22	5.09%

选项	小计	比例
一般	143	33.1%
符合	207	47.92%
完全符合	47	10.88%
**本题有效填写人次**	**432**	

第14题：数字音乐app的售后服务很大程度上影响着我是否会继续购买 [单选题]

选项	小计	比例
完全不符合	12	2.78%
不符合	18	4.17%
一般	119	27.55%
符合	212	49.07%
完全符合	71	16.44%
**本题有效填写人次**	**432**	

第15题：当数字音乐官方对待品牌出现的热点问题进行有效且合理的回应时，我会对该品牌的形象改观 [单选题]

选项	小计	比例
完全不符合	12	2.78%
不符合	14	3.24%
一般	144	33.33%
符合	219	50.69%
完全符合	43	9.95%
**本题有效填写人次**	**432**	

第16题：当数字音乐APP出现新的功能时，我会想要进行尝试 [单选题]

选项	小计	比例
完全不符合	15	3.47%
不符合	34	7.87%
一般	167	38.66%
符合	175	40.51%
完全符合	41	9.49%
**本题有效填写人次**	**432**	

第17题：当数字音乐APP征集改进意见时，去会积极进行反馈，以期之后得到更好的服务 [单选题]

选项	小计	比例
完全不符合	21	4.86%
不符合	47	10.88%
一般	214	49.54%
符合	120	27.78%
完全符合	30	6.94%
**本题有效填写人次**	**432**	

第18题：在选择购买数字音乐APP时，我会由于如个人对于封面设计等细节的审美而做出不同的选择 [单选题]

选项	小计	比例
完全不符合	14	3.24%
不符合	29	6.71%
一般	152	35.19%
符合	188	43.52%
完全符合	49	11.34%
**本题有效填写人次**	**432**	

第19题：在选择购买数字音乐APP时，我会参考大众的审美从而做出不同的选择 [单选题]

选项	小计	比例
完全不符合	14	3.24%
不符合	65	15.05%
一般	197	45.6%
符合	131	30.32%
完全符合	25	5.79%
**本题有效填写人次**	**432**	

第20题：在选择购买数字音乐时，相较于音质，我更在乎可获得数量 [单选题]

选项	小计	比例
完全不符合	40	9.26%
不符合	140	32.41%
一般	149	34.49%
符合	78	18.06%
完全符合	25	5.79%
**本题有效填写人次**	**432**	

第21题：在购买数字音乐时，相较于数量，我更在乎获得音质 [单选题]

选项	小计	比例
完全不符合	12	2.78%
不符合	19	4.4%
一般	152	35.19%
符合	175	40.51%
完全符合	74	17.13%
**本题有效填写人次**	**432**	

第22题：我会因为版权意识而倾向于更加正规、有资质的数字音乐APP [单选题]

选项	小计	比例
完全不符合	6	1.39%
不符合	19	4.4%
一般	143	33.1%
符合	196	45.37%
完全符合	68	15.74%
**本题有效填写人次**	**432**	

第23题：我会因为紧跟大众潮流而选择购买大众所推崇发数字音乐APP [单选题]

选项	小计	比例
完全不符合	23	5.32%
不符合	96	22.22%
一般	195	45.14%
符合	93	21.53%
完全符合	25	5.79%
**本题有效填写人次**	**432**	

第24题：我认为参与某个数字音乐APP的话题或购买，可以让我更好地融入社交圈子 [单选题]

选项	小计	比例
完全不符合	29	6.71%
不符合	92	21.3%
一般	178	41.2%
符合	111	25.69%
完全符合	22	5.09%

选项	小计	比例
本题有效填写人次	432	

第25题：我会向别人推荐购买我认为好用的数字音乐APP [单选题]

选项	小计	比例
完全不符合	13	3.01%
不符合	32	7.41%
一般	183	42.36%
符合	165	38.19%
完全符合	39	9.03%
本题有效填写人次	432	

第26题：我更倾向于持续购买大众眼中品牌形象好的数字音乐APP [单选题]

选项	小计	比例
完全不符合	12	2.78%
不符合	37	8.56%
一般	196	45.37%
符合	152	35.19%
完全符合	35	8.1%
本题有效填写人次	432	

第27题：当周边的亲戚、朋友、同学对数字音乐的选择开始转变时，我愿意尝试购买他们所推崇的新APP [单选题]

选项	小计	比例
完全不符合	16	3.7%
不符合	57	13.19%
一般	212	49.07%
符合	118	27.31%
完全符合	29	6.71%
本题有效填写人次	432	

第28题：我认为数字音乐APP投放的广告能够帮助我更好更精准的实现购买决策 [单选题]

选项	小计	比例
完全不符合	28	6.48%
不符合	84	19.44%

选项	小计	比例
一般	187	43.29%
符合	111	25.69%
完全符合	22	5.09%
**本题有效填写人次**	**432**	

第29题：单一明确的广告信息能够让我感受到数字音乐品牌的创意 [单选题]

选项	小计	比例
完全不符合	22	5.09%
不符合	56	12.96%
一般	195	45.14%
符合	131	30.32%
完全符合	28	6.48%
**本题有效填写人次**	**432**	

第30题：当身边的亲戚、朋友、同学向我推荐某一数字音乐APP时，我会优先考虑购买他们推荐的 [单选题]

选项	小计	比例
完全不符合	14	3.24%
不符合	55	12.73%
一般	197	45.6%
符合	142	32.87%
完全符合	24	5.56%
**本题有效填写人次**	**432**	

第31题：相较于不熟悉的数字音乐APP，我更倾向于购买被我熟知的 [单选题]

选项	小计	比例
完全不符合	7	1.62%
不符合	12	2.78%
一般	124	28.7%
符合	240	55.56%
完全符合	49	11.34%
**本题有效填写人次**	**432**	

第32题：数字音乐的活动宣传对我而言太过陌生(即我想要了解但品牌方宣传不到位) [单选题]

选项	小计	比例
完全不符合	8	1.85%
不符合	34	7.87%
一般	224	51.85%
符合	140	32.41%
完全符合	26	6.02%
**本题有效填写人次**	**432**	

第33题：我认为参与过音乐活动的人(热衷于音乐活动)更可能购买数字音乐 [单选题]

选项	小计	比例
完全不符合	10	2.31%
不符合	20	4.63%
一般	146	33.8%
符合	196	45.37%
完全符合	60	13.89%
**本题有效填写人次**	**432**	

第34题：相对于其他数字音乐APP，我更倾向于购买我参与过的主办音乐活动的品牌 [单选题]

选项	小计	比例
完全不符合	13	3.01%
不符合	27	6.25%
一般	176	40.74%
符合	180	41.67%
完全符合	36	8.33%
**本题有效填写人次**	**432**	

第35题：数字音乐品牌主办发活动能够让我产生共鸣 [单选题]

选项	小计	比例
完全不符合	16	3.7%
不符合	26	6.02%
一般	207	47.92%
符合	159	36.81%
完全符合	24	5.56%
**本题有效填写人次**	**432**	

第36题：数字音乐品牌举办的线上或线下音乐活动能够让我持续关注或想要参加 [单选题]

选项	小计	比例
完全不符合	18	4.17%
不符合	34	7.87%
一般	194	44.91%
符合	152	35.19%
完全符合	34	7.87%
**本题有效填写人次**	**432**	

第37题：当某数字音乐品牌出现热点时，我会选择参与其中(进行点赞评论转发) [单选题]

选项	小计	比例
完全不符合	23	5.32%
不符合	56	12.96%
一般	201	46.53%
符合	126	29.17%
完全符合	26	6.02%
**本题有效填写人次**	**432**	

第38题：在大学生涯中，我愿意利用生活费购买数字音乐 [单选题]

选项	小计	比例
完全不符合	28	6.48%
不符合	48	11.11%
一般	195	45.14%
符合	139	32.18%
完全符合	22	5.09%
**本题有效填写人次**	**432**	

第39题：我愿意推荐舍友、同学等购买数字音乐 [单选题]

选项	小计	比例
完全不符合	28	6.48%
不符合	73	16.9%
一般	204	47.22%
符合	105	24.31%
完全符合	22	5.09%

选项	小计	比例
**本题有效填写人次**	**432**	

第40题：通过渠道了解到数字音乐后对我购买数字音乐的购买欲望起到了促进作用 [单选题]

选项	小计	比例
完全不符合	23	5.32%
不符合	34	7.87%
一般	201	46.53%
符合	154	35.65%
完全符合	20	4.63%
**本题有效填写人次**	**432**	
